# Establishment of cell suspension culture in *Marchantia linearis* Lehm & Lindenb. for the optimum production of flavonoids

**DOI:** 10.1007/s13205-013-0123-7

**Published:** 2013-02-27

**Authors:** Remya Krishnan, V. S. Anil Kumar, K. Murugan

**Affiliations:** Plant Biochemistry and Molecular Biology Laboratory, Department of Botany, University College, Thiruvananthapuram, 695034 Kerala India

**Keywords:** Cell suspension, Culture parameters, Flavonoids, *Marchantia linearis*

## Abstract

Bryophytes are the second largest group in the plant kingdom, but studies conducted to better understand their chemical composition are limited and scattered. Axenically grown bryophytes expressed potential in biotechnological processes. The present study was designed to investigate the in vitro cell growth, culture parameters and their effect on flavonoid synthesis. Chlorophyll-containing callus cells of *Marchantia linearis* Lehm & Lindenb. is able to grow under low light in the presence of organic carbon source and retain the ability to produce flavonoids. Highest flavonoid production was achieved using 2,4-dichlorophenoxyacetic acid as growth hormone. Inoculum size, light intensity, organic carbon source and cations are the culture parameters affecting flavonoid productivity. Maximum flavonoid productivity is observed under low light intensity, with a photon flux density ca. 20 μmol/m^2^/s. Optimal inoculum size and glucose concentration for flavonoid production are 10–14 and 2–3 %, respectively. Cations like ferrous trigger flavonoid synthesis by increasing its intracellular concentrations. Flavonoid production in the cell culture is shown to be significantly growth related. Osmotic stress is ineffective in triggering flavonoid synthesis. Methyl jasmonate and 2-(2-fluoro-6-nitrobenzylsulfanyl) pyridine-4-carbothioamide elicitors showed positive effect on intracellular flavonoid content in cultured cells. Using the standard plot of quercetin (*y* = 0.0148*x*, *R*^2^ = 0.975), the flavonoid contents of in vitro samples were found ranging from 4.0 to 17.7 mg quercetin equivalent/g tissue. Flavonoids are fractionated by HPLC-PAD revealed the presence of quercetin (182.5 μg/g), luteolin (464.5 μg/g) and apigenin (297.5 μg/g). Further studies are warranted to analyze the therapeutic potentiality of the flavonoids in the liverwort.

## Introduction

Flavonoids are a large group of low molecular weight, ubiquitously distributed, polyphenolic secondary metabolites. These compounds play a significant role in various stages of plant growth and their existence in the environmental stresses. Flavonoids act as signal molecules to take preventive measures to save them from pathogenic microbial attack (Harbone and Willians 2000). Flavonoids are important for human beings due to their antioxidative and radical scavenging effects as well as their potential estrogenic and anticancer activities. Over 5,000 naturally occurring flavonoids have been characterized from higher plants (Jedinak et al. 2004). Plant cell cultures are viewed as an attractive source for the production of biologically active compounds as a source of natural food health supplements, which is of importance because of the restrictions imposed on synthetic supplements (Tascan and Adelberg 2010). Although culturing plant tissues and organs under axenic conditions was first established and profitably employed in bryophytes, especially mosses, bryophytes did not retain for long their rightful place as a highly favoured research object; therefore, most studies of plant morphogenesis are now being done on vascular plants. Besides the problems with bryophyte establishment in axenic culture, there also exist some problems of material availability, genetic variability of material, disposal of axenic organisms leaving on bryophytes and low level of species biology knowledge. *Marchantia polymorpha* is the most intensely studied bryophyte (Kaul et al. 1962). Vujicic et al. (2011) developed a protocol to isolate secondary metabolites from axenic cultures of bryophytes. Previously, Decker and Reski (2008) have established moss bioreactors for improved biopharmaceuticals. The major identified phytoconstituents are flavonoids, triterpenoids and steroids (Toyota et al. 2004; Niu et al. 2006). The flavonoids characterized in liverworts are mainly quercetin, luteolin, apigenin and their O- and C-glycosides. Calli of *Marchantia* species form fast-growing cell suspension cultures and are regarded as the most suitable in vitro culture to synthesize flavonoids under photo-mixotrophic growth conditions compared to other bryophytes (Kaul et al. 1962). In this study, we have focused to investigate the in vitro cell growth, the culture parameters and their effect on flavonoid synthesis in shake flask cultures of *Marchantia linearis* Lehm & Lindenb. under photomixotrophic growth conditions. Subsequently, the total flavonoids were quantified spectrophotometrically and its fractionation by HPLC-PAD method.

## Materials and methods

### Plant material

Fresh thallus of *Marchantia linearis* Lehm & Lindenb. was collected from the Kallar river floor of Ponmudi Hills, Thiruvananthapuram, Kerala, India. Taxonomic identity was confirmed by comparing with authenticated herbarium specimen at Department of Botany Herbaria, University of Calicut, Kerala. A voucher specimen of the plant was kept in the herbarium of the institute (UC DB 324).

### Establishment of axenic in vitro callus cultures

Fresh spores of *M. linearis* were collected from a single sporogon of a haploid thallus. Spore surfaces were sterilized with 0.5 mL 1 % freshly prepared calcium hypochlorite solution for 5 min, washed with 5 × 1 mL sterile H_2_O and centrifuged, and stored in sterile H_2_O at 7 °C. Spores germinated within 3–4 weeks at 15 °C on a modified Knop agar (10 mL) supplemented with 10 μL ampicillin (1,000 U/mL), 10 μL amphotericin B (250 μg/mL), and 10 μL nystatin (1,000 U/mL). Single plantlets (approximately 500 μm in diameter) were transferred to petridishes with modified Knop agar (10 mL) supplemented with above-mentioned antibiotics and cultivated at 15 °C. After sub-culturing, antibiotics were withdrawn and axenic conditions were regularly checked by transferring to liquid broth agar and potato dextrose agar test media at 23 °C for a week. Agar subcultures were kept in petridishes sealed with Nescofilm^®^ to avoid contamination and rapid drying.

### Cell suspension culture conditions

To establish suspension cultures, calli were first transferred into the liquid MSK-2 medium (mg/L) that contains NH_4_NO_3_, 1,650; KNO_3_, 1,900; CaCl_2·_2H_2_O, 440; MgSO_4·_7H_2_O, 370; KH_2_PO_4_, 216; KI, 0.83; H_3_BO_3_, 6.2; Mn-SO_4·_4H_2_O, 22.3; ZnSO_4·_7H_2_O, 8.6; Na_2_Mo-O_4·_2H_2_O, 0.5; CuSO_4·_5H_2_O, 0.05; CoCl_2·_6H_2_O, 0.05; FeSO_4·_7H_2_O, 26; Na_2_EDTA_·_2H_2_O, 37.3; *myo*-inositol, 90; nicotinic acid, 1; pyridoxine–HCl, 0.5; thiamine-HCl, 1; rhamnose, 2.25; biotin, 0.01; malic acid, 40.2; cyanocobalamine, 0.02; glucose, 20,000 and 2,4-dichlorophenoxyacetic acid (2,4-D), solidified with 0.8 % agar (pH 5.8) (Katoh et al. 1980). Cells were sub-cultured every 3 weeks at 25 °C and with photon flux density of 18 μmol/m^2^/s with 16:8-h light–dark photo-period. Fine cell clumps of average diameter ca. 0.5 mm harvested during exponential growth phase were used as inoculums. Fast-growing cells were suspended in 50 mL medium in 250 mL conical flasks and cultured on a rotary shaker (100 rev/min) at 25 °C under illumination by fluorescent lamps using a 16:8-h light–dark photo-period. Inoculum size and light intensity were denoted as in the results. Productivity (in mg/L/day) was calculated as the final yield of flavonoid (mg/L) divided by the total culture period (in days). Culturing was terminated as soon as the glucose in the medium was exhausted. Culture experiments were carried out in triplicates. Data were denoted as mean ± SD.

### Other analytical methods

#### Reverse phase high performance liquid chromatography (RP-HPLC) PAD of flavonoids

The chromatographic system (Waters Company) consisted of Millennium 32 system software, Waters 717 plus Autosampler, Model Waters Delta 600 pump, and Model Waters 2996 Photodiode Array Detector. Chromatographic separation was carried out by HIQ SIL C_18_V reversed-phase column (4.6 mm Ф × 250 mm) packed with 5 μm diameter particles, the mobile phase is methanol–acetonitrile–acetic acid–phosphoric acid–water (200:100:10:10:200, V/V). The mobile phase was filtered through a 0.45 μm membrane filter (Millipore), and then deaerated ultrasonically prior to use. Flavonoids such as quercetin, (Q) luteolin (L), apigenin (A) were quantified by a PAD following RP-HPLC separation at 254.5 nm for Q, 345 nm for L and A. The flow rate was 1 mL/min, the injection volume was 25 μL and the column temperature was maintained at 30 °C. The chromatographic peaks of the analytes were confirmed by comparing their retention times and UV spectra with those of the reference standards. Quantification was carried out by the integration of the peak using external standard method.

#### Measurement of cell growth

For cell growth measurement, cell suspension was poured into a graduated test tube attached to the culturing vessel. Ratio of cell volume to total volume was read after sedimentation, denoted as % settled cell volume (SCV). Cell dry weight was measured by suction-filtering of the cell suspension with a filter paper and lyophilizing for 24 h.

#### Measurement of glucose residue

Glucose concentration in the culture medium was monitored as a growth parameter using Sigma diagnostic kit by conversion of glucose with peroxidase and glucose oxidase in the presence of *o*-dianisidine to a brown pigment, which was then, converted into a pink dye (485 nm) by addition of 2.5 mL 30 % H_2_SO_4_.

#### Determination of total flavonoid content

Various in vitro cell suspension cultures (100 g) were extracted with 300 mL of ethanol for 12 h using soxhlet hot extraction method. The supernatants were concentrated using rotavapor at 50 °C. The yield of the extract was 12.7 g. The residues were lyophilized and stored at −20 °C. Total flavonoid content was quantified by aluminum chloride method (Mervat et al. 2009). Aliquot of extract was made up to 3 mL using methanol. Further, 0.1 mL AlCl_3_ (10 %), 0.1 mL Na–K tartrate and 2.8 mL distilled water were added sequentially and the test solution was vigorously shaken. Absorbance was recorded at 415 nm after 30 min of incubation. A standard calibration plot was generated using known concentrations of quercetin. The concentrations of flavonoid in the samples were calculated from the calibration plot and expressed as mg quercetin equivalent/g of sample.

### Statistical analysis

Each data point is the mean of three replicates obtained from 3 to 5 independent experiments. All experimental data were analyzed by an analysis of variance (ANOVA). After confirming the significance of *F* values, the significance of differences between the mean values was tested using ANOVA. Significant differences were considered at *P* < 0.01 probability levels.

## Results and discussion

### Fractionation and quantification of flavonoids

**Fig. 1:**
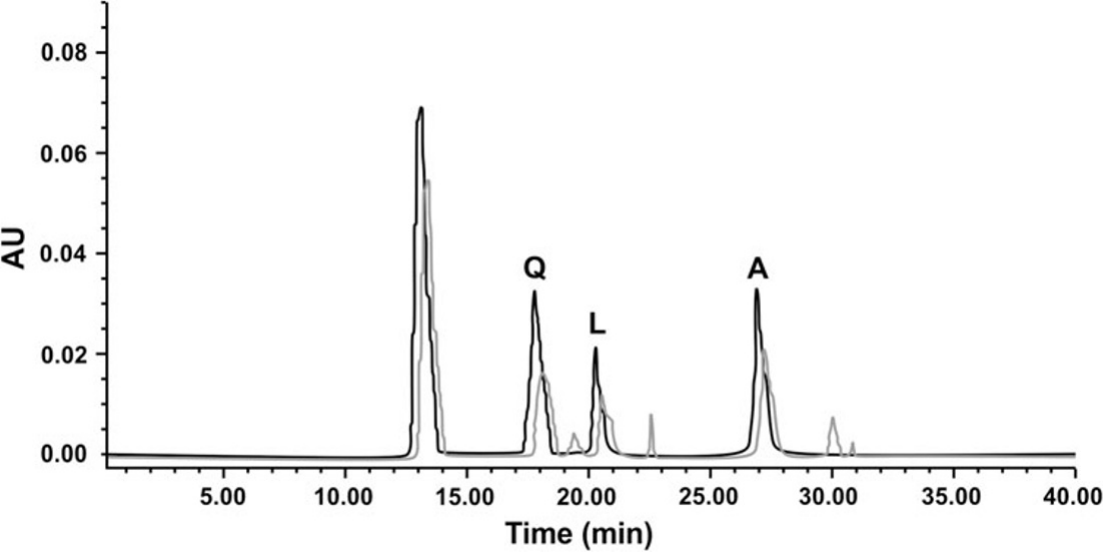
HPLC-PAD chromatogram of flavonoids of standards cospurged with *M. linearis* extract: (A) *Q*; (B) *L*; (C) *A*. Column: HIQ SIL C_18_V. Mobile phase: methanol–acetonitrile–acetic acid–phosphoric acid–water (200:100:10:10:200, v/v/v). Flow rate: 1 mL/min. Photodiode array detector at 254.5 nm for (A), 345 nm for (B) (*dark straight line* Standard, *light straight lineM. linearis* extract)

Extraction of flavonoids with 80 % ethanol under the frequency of 100 kHz, the temperature of 25 °C, the liquid–solid ratio of 10 mL/g and the time of 15 min, repeated thrice give the highest flavonoid yield (Fig. 1). Good results were obtained with respect to repeatability relative standard deviation (RSD) and recovery (97.3–99.6 %). The contents of quercetin, luteolin and apigenin in *M. linearis* are 182.5, 464.5 and 297.5 μg/g, respectively. Data are the average ± SD of three independent measurements. The fractionated flavonoid content in *M. linearis* is comparable with *M. polymorpha* (Zhu et al. 2004).

### Time course of cell growth and flavonoid production in shake flasks

**Fig. 2:**
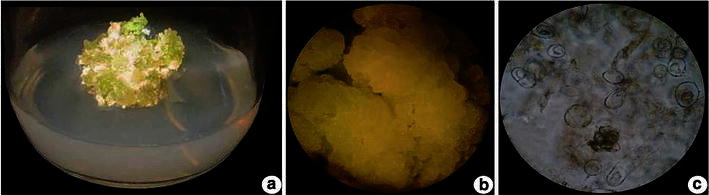
Culture of *M. linearis*—**a** callus, **b** cell suspension, and **c** cells enlarged

Table 1 displays a typical time course of flavonoid production in *M. linearis* suspension culture in the MSK-2 medium. A positive correlation was observed between flavonoid content and cell growth (Fig. 2a–c). After 10 days of culturing, the cells entered the late exponential or early stationary phase and flavonoid content reached its maximum. In this experiment, 4 % culture inoculum was used, and the maximum flavonoid content attained was 11.2 mg/L. The instantaneous productivity of flavonoid (mg/L/day) is not a constant during the course of the culture. As indicated in Table 1, maximum productivity is seen during the exponential growth phase (day 12–16). For comparative analysis, the productivity data reported in this study were the mean value ± SD based on the entire culture cycle and was statistically significant (*P* < 0.01). In all experiments, the culture was terminated as soon as glucose in the medium was exhausted.

**Table 1:** Effect of cell growth and flavonoid productivity in cell suspension culture

Culturing time (days)	Biomass (g^−1^)	SCV (%)	Flavonoid (mg/L/day)
0	0	0	0
2	1.2 ± 0.01	5 ± 0.1	2.0 ± 1.2
4	3.8 ± 0.1	6 ± 0.3	2.2 ± 1.7
6	7.8 ± 0.3	10 ± 0.12	6.0 ± 1
8	11.2 ± 0.25	12 ± 0.22	7.85 ± 2
10	12.6 ± 0.4	13 ± 0.2	9.49 ± 2.4
12	12.6 ± 0.3	14 ± 0.12	9.76 ± 1.8
14	12.6 ± 0.1	15 ± 0.7	10.7 ± 1.4
16	12.8 ± 0.2	15 ± 0.7	11.2 ± 0.2

Data are the mean ± SD of three independent measurements

Significant at *P* < 0.01. *F*-ratio = 0.82**, CD_(0.05)_ = 1.429

### Effect of carbon source, growth regulators, light intensity, inoculum size, cations and environmental stress on cell growth and flavonoid synthesis

Carbon source is known to affect a range of culture parameters such as growth, primary metabolism and yield of secondary products. In general, growth rate is considered as a function of carbon concentration. Highest cell growth and flavonoid production are obtained from medium supplemented with glucose followed by fructose, galactose and sucrose (Table 2). The presence of organic carbon source is necessary for cell growth; however, high sugar concentration leads to an increase in osmotic pressure that inhibits cell growth and thereby reducing flavonoid synthesis. Phenolic acids such as coumarate + glucose or cinnamate + glucose in the culture medium showed browning (i.e. loss of chlorophyll) and subsequent cell death, suggesting that the cells were unable to carry out conversion of phenolic acids into flavonoids. When mannitol was supplemented to the glucose containing medium, cell growth (retained green) was retarded, subsequently, the flavonoid content. The combined effect of the variables had a strong effect on the yield, *R*^2^ = 0.098, and was statistically significant (*P* < 0.01).

**Table 2:** Effect of carbon source on productivity of biomass and flavonoid content

Carbon source (2 % W/V)	Productivity (mg/L/day)	
	Biomass (g^−1^)	Flavonoid
Galactose	598.4 ± 0.89	8.4 ± 0.1
Fructose	610.6 ± 1.2	9.5 ± 0.7
Glucose	864 ± 6.8	12.4 ± 1
Glucose/cinnamate^a^	–	–
Phenylalanine	386.4 ± 2.7	4.9 ± 0.2
Lactose	20.5 ± 0.01	0.78 ± 0.01
Maltose	330 ± 5.2	1.8 ± 0.4
Mannitol	10.6 ± 0.02	0.5 ± 0.01
Glucose/coumarate^a^	–	–
Sucrose	700.2 ± 6.9	7.3 ± 0.5

Data are the mean ± SD of three independent measurements

Significant at *P* < 0.01. *F*-ratio = 3.569**, CD_(0.05)_ = 2.76

^a^Cells are found dead on 3rd day

The growth of chlorophyll-containing cells in media supplemented with different amount of glucose was compared (Table 3). Cells cultured in medium without supplement of glucose retained their viability (appeared green) but showed poor proliferation. Biomass and flavonoid synthesis peaked at a glucose concentration of 2–3 %. Further increase of glucose concentration resulted in a reduction of flavonoid content remarkably than that of biomass. The strengths of the medium and concentrations of glucose played crucial roles in the growth rate and the data were significant (*P* < 0.01).

**Table 3:** Effect of glucose concentration on productivity of biomass and flavonoid content

Glucose concentration (% W/V)	Productivity (mg/L/day)	
	Biomass (g^−1^)	Flavonoid
0	60.4 ± 1.9	0.8 ± 0.01
1	798 ± 10.2	6.7 ± 0.5
2	1080 ± 8.9	9.4 ± 0.3
3	1260 ± 7.8	11.9 ± 1.2
4	1092 ± 6.5	8.2 ± 0.1
5	918.5 ± 1.7	5.3 ± 0.1

Data are the mean ± SD of three independent measurements

Significant at *P* < 0.01. *F*-ratio = 11.54**, CD_(0.05)_ = 2.433

Medium supplemented with various combination of growth hormones analyzed includes BAP (1 mg/L), 2,4-D (0.5 mg/L), kinetin (0.5 mg/L) or in combination with 2,4-D (0.5 mg/L) + BAP (1 mg/L), 2,4-D (0.5 mg/L) + kinetin (0.5 mg/L) or NAA (0.5 mg/L) or NAA (0.5 mg/L) + BAP (1 mg/L), or NAA (0.5 mg/L) + Kin(0.5 mg/L). Initially, cells were acclimatized to the medium containing the standard growth hormones for 6 months before growth and productivity measurements were attempted (Table 4). Interestingly, high flavonoid productivity does not always correspond to high biomass yield. Highest flavonoid productivity was obtained in cultures with 2,4-D at 12.8 ± 0.3 mg/L/day, as well as with 2,4-D + BAP was 10.5 ± 0.2 mg/L/day. The results suggest that 2,4-D was the ideal hormone for cultures considered for further studies. The corresponding *P* and *R*^2^ values were *P* < 0.001 and 0.097, respectively.

**Table 4:** Effect of growth regulators on productivity of biomass and flavonoid content

Growth regulator	Productivity (mg/L/day)	
	Biomass (g^−1^)	Flavonoid
Control	614.5 ± 3.9	2.4 ± 0.2
BAP (1 mg/L)	362.8 ± 2.4	3.6 ± 0.3
Kin (0.5 mg/L)	510.4 ± 0.9	5 ± 0.2
2,4-D (0.5 mg/L)	1036.5 ± 1.7	12.8 ± 0.3
2,4-D (0.5 mg/L) + BAP (1 mg/L)	1100 ± 3.5	10.5 ± 0.2
2,4-D (0.5 mg/L) + Kin (0.5 mg/L)	498 ± 0.1	4 ± 0.3
NAA (0.5 mg/L)	684 ± 9.2	5.7 ± 1
NAA (0.5 mg/L) + BAP (1 mg/L)	1200.5 ± 1.4	6.7 ± 0.32
NAA (0.5 mg/L) + Kin (0.5 mg/L)	1090 ± 7.3	8.3 ± 0.6

Data are the mean ± SD of three independent measurements

Significant at *P* < 0.01. *F*-ratio = 0.597**, CD_(0.05)_ = 3.554

*Marchantia* cells were able to grow in the dark, as well as under intense light, with glucose as the carbon source (Su and Chiou 1998). No significant changes were noted under different light intensities related to growth rate and biomass yield. However, intracellular flavonoid attained maximal level at a photon flux density around 20 μmol/m^2^/s is 12 ± 0.2 mg/L/day, which is about 1.6 % higher than that at 3 μmol/m^2^/s (i.e. 7.5 ± 0.2 mg/L/day). The change in flavonoid yield is mainly a result of the increase of phenolic acid content, rather than changes of biomass yield (Table 5).

**Table 5:** Effect of photodensity on productivity of biomass and flavonoid content

Productivity (mg/L/day)	Photon flux density (μmol/m^2^/s)			
	3	9	20	32
Biomass	1010 ± 1.4	960 ± 7.4	951 ± 1.7	950 ± 2.8
Flavonoid	7.5 ± 0.2	9.8 ± 0.5	12 ± 0.2	10.4 ± 0.9

Data are the mean ± SD of three independent measurements

Significant at *P* < 0.01. *F*-ratio = 10.88**, CD_(0.05)_ = 4.845

Inoculum size (%) showed a positive correlation with flavonoid yield and biomass production even in short culture period (Table 6). As inoculum size increased to 8 %, biomass and flavonoid productivity reached 1117 ± 2.4 mg/L/day and 10 ± 0.4, respectively; both were much higher than that with 2 % inoculum size. Further enhancement on flavonoid productivity was noticed by increasing the inoculum size to 12 % (flavonoid level 12.8 ± 0.4 mg/L/day). However, the flavonoid productivity decreased marginally with higher inoculum size 14 % (12 ± 0.9 mg/L/day) with change in biomass (1182 ± 1.2 mg/L/day). Inoculum size affected productivity by shortening the lag phase, thus, increasing the culture efficiency.

**Table 6:** Effect of inoculums size on productivity of biomass and flavonoid content

Inoculums (%)	Productivity (mg/L/day)	
	Biomass (g^−1^)	Flavonoid
2	440 ± 4.3	4 ± 0.5
4	816 ± 7.2	5.2 ± 0.4
8	922 ± 1.3	7.3 ± 0.4
6	1100 ± 2.4	9.5 ± 0.4
8	1117 ± 2.4	10 ± 0.4
10	1142 ± 1.3	10.9 ± 0.3
12	1176 ± 2.1	12.8 ± 0.4
14	1182 ± 1.2	12.0 ± 0.9

Data are the mean ± SD of three independent measurements

Significant at *P* < 0.01. *F*-ratio = 5.16**, CD_(0.05)_ = 11.05

Addition of cations such as Mg^2+^, Mn^2+^, Cu^2+^ and Ca^2+^ to the regular culture medium as sulfate salts showed no remarkable change in biomass production. However, productivity of flavonoid was increased with Fe^2+^ to 12 ± 1.2 mg/L/day (Table 7). Flavonoids are free radical scavengers against oxygen radicals and also chelators against Cu^2+^ (Park et al. 1991). But in runner bean plants, particularly in young stage Cd^2+^ and Cu^2+^ stress induced flavonoid synthesis (Skorzynska-Polit et al. 2004).

**Table 7:** Effect of cations on productivity of biomass and flavonoid content

Cations (30 % increase)	Productivity (mg/L/day)	
	Biomass (g^−1^)	Flavonoid
Control	1160 ± 3.1	2.6 ± 0.2
Mg^2+^	1147 ± 2.3	5.8 ± 0.2
Mn^2+^	1032 ± 1.4	7.4 ± 0.4
Cu^2+^	1155 ± 1.6	8 ± 0.5
Fe^2+^	1200 ± 1.3	12 ± 1.2
Ca^2+^	1156 ± 0.4	9 ± 0.42

Data are the mean ± SD of three independent measurements

Significant at *P* < 0.01. *F*-ratio = 5.89**, CD_(0.05)_ = 2.417

Similarly, no remarkable change in flavonoid content was observed by changing the concentration of major and minor nutrients of the medium. Environmental stresses such as osmotic stress, resulting from addition of NaCl or mannitol, decreased the flavonoid productivity (Table 8). Growth inhibition was noted with NaCl (0.2–0.5 %) and sucrose concentration (2–6 %). Stoynova-Bakalova et al. (2009) revealed that environmental stress significantly regulates the synthesis of polyphenols. Variation in metabolites can be linked to ontogenetic transformation of plant metabolism.

**Table 8:** Effect of osmolarity on productivity of biomass and flavonoid content

Osmolarity	Productivity (mg/L/day)	
	Biomass (g^−1^)	Flavonoid
NaCl (%)		
0	560 ± 1.4	4.5 ± 0.2
0.1	647 ± 1.5	7.8 ± 0.3
0.2	422 ± 0.5	3.5 ± 0.01
0.5	200 ± 0.21	1.5 ± 0.05
Sucrose (%)		
0	1060 ± 1.2	3.8 ± 0.9
2	800 ± 0.3	5.7 ± 0.4
4	510 ± 1.2	5.3 ± 0.1
6	260 ± 0.5	1.7 ± 0.1

Data are the mean ± SD of three independent measurements

Significant at *P* < 0. 01. *F*-ratio = 12.07**, CD_(0.05)_ = 22.65

Methyl jasmonate and 2-(2-fluoro-6-nitrobenzylsulfanyl) pyridine-4-carbothioamide, which have been commonly used as elicitors in stimulating secondary metabolite production, were also evaluated. Both these elicitors induced intracellular flavonoid level (Table 9). Methyl jasmonate stimulated cotyledon growth coupled with kaempferol, rhamnoside content, but reduced rutin level in *Cucurbita pepo* (Stoynova-Bakalova et al. 2009). Synthetic elicitor, 2-(2-fluoro-6-nitrobenzylsulfanyl) pyridine-4-carbothioamide, showed the best elicitation effect after 48 h application of 1 μmol/L concentration and the data were statistically significant at *P* < 0.001. The present results are comparable with that of suspension culture of *Trifolium pratense* (Kasparov et al. 2012).

**Table 9:** Effect of methyl jasmonate and 2-(2-fluoro-6-nitrobenzylsulfanyl) pyridine-4-carbothioamide on productivity of biomass and flavonoid content

Elicitors	Productivity (mg/L/day)	
	Biomass (g^−1^)	Flavonoid
Methyl jasmonate		
Control	1012 ± 0.2	6.7 ± 0.44
100 μM	1240 ± 0.6	13.5 ± 0.27
200 μM	1308 ± 0.2	16.4 ± 0.08
2-(2-fluoro-6-nitrobenzylsulfanyl) pyridine-4-carbothioamide		
0	1011 ± 0.4	5.8 ± 0.6
1 μmol/L	1297 ± 0.6	15.4 ± 0.3
10 μmol/L	1370 ± 0.8	17.7 ± 0.2

Data are the mean ± SD of three independent measurements

Significant at *P* < 0.01. *F*-ratio = 0.458**, CD_(0.05)_ = 13.09

Previous in vitro culture studies have showed no callus growth in dark even in the presence of organic carbon (Katoh et al. 1980; Ohta et al. 1977). Interestingly, in the present study, cultured *M. linearis* cells were found capable of growing under low light at a rate comparable with high light intensity. Apparently, although cultured *M. linearis* cells contain chlorophyll, their photosynthetic capacity is low.

**Fig. 3:**
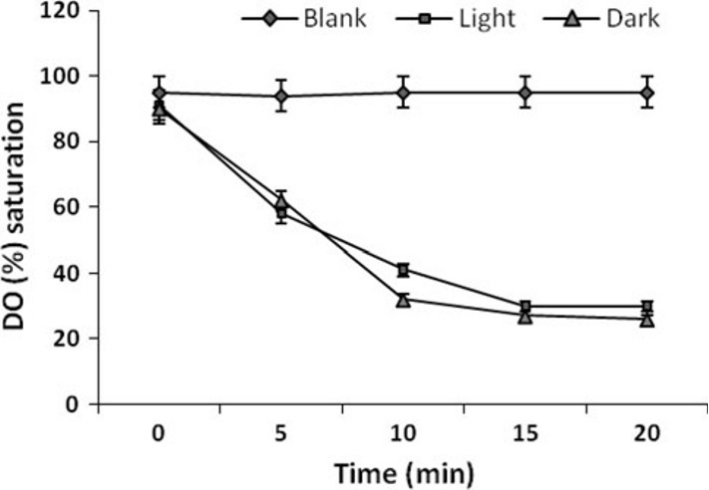
Comparison of DO value in cell suspension culture of *M. linearis* under illumination or in dark. Photon of intensity was 610 μmol/m/s. Cells are tested in a vessel of i.d. 2.6 cm

To study the photosynthetic efficiency of the *M. linearis* culture, oxygen consumption of the culture under light versus dark conditions was compared using early exponential phase cells. In this experiment, cells were suspended in fresh culture medium to a concentration of 6 g dry wt/L, placed in a vessel, and agitated using a small magnetic stirring bar. The vessel was either illuminated with two lamps on opposite sides of the vessel at a photon flux density of 610 μmol/m^2^/s (measured on the outer vessel surface) or was shielded from light. Dissolved oxygen (DO) was measured with an oxygen electrode at 25 °C and represented as percentage of air saturation. Before starting the DO recording, the medium was saturated with air. DO profile during the measurement (at 25 °C) is shown in Fig. 3. The variation in the DO concentration in the illuminated culture is a result of both oxygen consumption (respiration) and oxygen evolution (photosynthesis). Under illumination, DO of cell suspension was found to decrease from 92 to 35 % in 20 min. In the dark, the DO decreased from 88 to 27 % during the same period. The DO of cell-free medium (blank) remained at ca. 90 % throughout the measurement. The apparent O_2_ consumption rate was calculated to be 0.3651 and 0.3907 μmol/mg/h for light and dark conditions, respectively, suggesting low photosynthetic capacity under light. CO_2_, a byproduct of respiration, its dissolution in water is much higher than that of oxygen and, therefore, it is not a limiting factor for photosynthesis. Thus, it is possible to suggest that the low photosynthesis efficiency in the cells was due to poor photosynthetic capacity rather than shortage of CO_2_. Cultured *M. linearis* cells derived their energy mainly through the consumption of organic carbon source. Large-scale proliferation of *M. linearis* cells is hence possible under heterotrophic growth conditions with low or no illumination, although with a slightly lower flavonoid content.

From a scale-up point of view, phototrophic growth is not as desirable as heterotrophic growth considering the difficulty in efficient large-scale bioreactor illumination. Modification on bioreactor design could partially compensate for the problem in light source. This could be done by increasing the reactor surface to volume ratio as in the case of tubular photo bioreactors (Molina Grima et al. 1994), or by modifying the light delivery system using, for instance, optical fibers (Burgess et al. 1993). These modifications nonetheless increase the cost and complexity of scale-up.

Culture temperature is another parameter which could affect flavonoid synthesis. Suspension culture of *M. linearis* was unable to grow at temperatures ≤37 °C. The results revealed that 25 °C was optimal than 15 °C on flavonoid production. Similarly, culture temperature also affected growth rate but not flavonoid yield. In addition, low temperature significantly increased the culture time i.e. 7 days at 25 °C and 18 days at 15 °C. According to these results, flavonoid productivity was calculated to be 13.8 and 6.1 mg/L/day for 25 and 15 °C, respectively. Therefore, 25 °C was fixed to study other culture factors affecting flavonoid synthesis.

In the present study, flavonoid productivity is mostly at par with biomass density during the culture period. The relationship between flavonoid and biomass indicated that flavonoid production is growth related. In this juncture, it may be desirable to use high-density culture as an alternative strategy to enhance flavonoid productivity. To achieve high biomass density, fed batch culture or perfusion culture with constant cell harvesting may lead to further improvement of flavonoid production using *M. linearis* cell suspension culture (Su 1995).

Bryophyte differs from higher plants in their ability to synthesize flavonoid in cell culture techniques. Jiang et al. (2006) cloned and characterized chalcone synthase from the bryophyta, *Physcomitrella patens.* Upman and Sarin (2011) reported flavonoid production in calli of *Indigofera* i.e. maximum total flavonoids were observed in 6 weeks old callus culture (1.55 mg/g dw) and minimum at 2 weeks old callus culture (0.87 mg/g dw). Similarly, Tascan and Adelberg (2010) observed a similar trend in *Scutellaria lateriflora* in vitro culture. It is obvious from the data that flavonoid synthesis of higher plants differs from that of bryophytes. Several studies on in vitro culture of bryophytes have been reported (Takio et al. 1986; Ono et al. 1987; Hansen and Rossi 1991). However, the relation between flavonoid production and growth/biomass yield was scarce. Proliferation efficiency is, however, one of the major factors to be considered while applying plant cell culture for flavonoid production. Similarly, successful elicitation depends on many factors that are specific for each plant. Elicitation strengthens the transcription of genes related to the secondary metabolic pathway and thus, genes that code enzymes which are necessary for synthesis of isoflavonoids and flavonoids (phenylalanine ammonia lyase, chalcone synthase (Jiang et al. 2006). 2-(2-fluoro-6-nitrobenzylsulfanyl) pyridine-4-carbothioamide, has shown to be an effective elicitor of phenylpropane metabolism (Kasparov et al. 2012).

*M. linearis* was reported to grow photoautotrophically in a CO_2_-enriched (1 %) atmosphere (Shinmen et al. 1991). However, higher growth rate and flavonoid productivity can be obtained in photomixotrophic growth than in photoautotrophic growth. Furthermore, under optimal culture conditions with sufficient illumination (20 μmol/m^2^/s) and proper inoculum size (8–12 %), productivity of photomixotrophic culture in the present study is higher. The productivity in shake flask culture could be further improved for flavonoid in Fe^2+^ enriched culture. The present results suggest the possibility of flavonoid production with *M. linearis* using shake flask culture without CO_2_ enrichment, which meant cells, can utilize an organic carbon source as their energy supplement to produce flavonoid. The results of the present investigation of the effect of elicitors on secondary metabolism may provide greater insight into the physiological significance of the induction of new proteins and activation of gene expression. Further studies are warranted to link the genome with flavonoid synthesis.
